# Multisystem ALK-positive histiocytosis: a multi-case study and literature review

**DOI:** 10.1186/s13023-023-02649-x

**Published:** 2023-03-13

**Authors:** Wei Liu, Hong-jie Liu, Wei-ya Wang, Yuan Tang, Sha Zhao, Wen-yan Zhang, Jia-qi Yan, Wei-ping Liu

**Affiliations:** 1 Department of Pathology, Fujian Medical University Cancer Hospital, Fujian Cancer Hospital, Fuzhou, China; 2 Department of Dermatovenereology, West China Hospital, Sichuan University, Chengdu, China; 3 Department of Pathology, West China Hospital, Sichuan University, No. 37 Guo Xue Xiang, Chengdu, 610041 Sichuan Province China

**Keywords:** ALK-positive histiocytosis, KIF5B-ALK, EML4-ALK, Multisystem involvement

## Abstract

**Background:**

Anaplastic lymphoma kinase (ALK)-positive histiocytosis, a novel rare histiocytic proliferation, was first described in 2008; it occurs in early infancy with liver and hematopoietic involvement. The spectrum was subsequently broadened to include localized diseases in older children and young adults. However, its full clinicopathological features and molecular lineage have not been fully elucidated.

**Results:**

Here, we report four cases of multisystem ALK-positive histiocytosis without hematopoietic involvement. Clinically, three patients were adults aged between 32 and 51 years. Two patients’, whose main manifestations were intracranial mass and numerous micronodules in the thoracoabdominal cavity organs and skin papules respectively, had a partial response to ALK inhibitors after surgery. One patient presented with mediastinal neoplasm without surgical treatment, and progressive disease occurred after two years of ALK inhibitor therapy. The fourth patient was a 17-month-old male with a large intracranial mass and presented with a poor response to ALK inhibitor and chemoradiotherapy; he died eight months after surgery. Pathologically, the histiocytes were large, with abundant eosinophilic cytoplasm, and mixed with variable numbers of foamy cells and Touton giant cells. Interstitial fibrosis was also observed. Histiocytes were positive for macrophage markers (CD68 and CD163) and ALK. *KIF5B-ALK* fusions were detected in two cases, *EML4-ALK* in one, and both *DCTN1-ALK* and *VRK2-ALK* fusions were detected in one case.

**Conclusions:**

We observed that ALK inhibitors present robust and durable responses in adult patients but a poor response in young children with central nervous system involvement. There is no consensus on the optimal treatment regimen and long-term prognosis requires further observation. Moreover, every unusual histiocytic proliferative lesion, especially unresectable and multisystem involvement, should be routinely tested for ALK immunohistochemical staining to identify this rare disease.

**Supplementary Information:**

The online version contains supplementary material available at 10.1186/s13023-023-02649-x.

## Background

Anaplastic lymphoma kinase (ALK)-positive histiocytosis, a novel systemic histiocytic proliferation in early infancy with hepatosplenomegaly and hematological abnormalities, was first described in 2008 [1]. The researchers subsequently reported seven additional cases, including two infants and one little child with similar systemic involvement and four cases with localized lesions in older children and young adults, which expanded the clinicopathological spectrum of the disease. The previous study showed that *KIF5B-ALK* was the most common gene fusion [2]. Moreover, ALK-positive histiocytosis has been considered a distinct histiocytic neoplasm in the 5th WHO classification and International Consensus Classification [3, 4].

Histological manifestations were diverse; histiocytes are usually very large, with irregularly folded nuclei, fine chromatin, and abundant eosinophilic cytoplasm [1, 2]. However, some cases display fascicular and storiform growth of uniform spindle cells admixed with lymphocytic infiltrates in the background, which might be easily misdiagnosed as inflammatory disease, or other proliferative lesions, such as inflammatory myofibroblastic tumors (IMT) [5, 6].

These data suggest that ALK-positive histiocytosis is rare and highly heterogeneous, including the age of onset, clinical manifestations, and histology, and its full clinicopathological and molecular features are not fully understood. It is necessary to describe more cases to elucidate the nature of this disease. Here, we report four cases of multisystem ALK-positive histiocytosis without hematopoietic involvement. We believe that this study will aid clinicians and pathologists in better identification of this rare entity.

## Results

The clinical, pathological, and molecular findings are summarized in Table 1, and detailed molecular data are provided in Additional file 1.

**Table 1:** Clinicopathological features of ALK-positive histiocytosis

Subjects	Case 1	Case 2	Case 3	Case 4
*Clinical features*				
Gender/Age	M/38 y	F/51 y	M/32 y	M/17 mo
Organ involved	Lung, mediastinum, LN	Brain, lung, LN	Liver, gallbladder, skin, lung, pancreas, kidney, LN	Brain, lung, liver, skin
Size of major lesions	Lung, 5.5 × 5 cm	Brain, 2.5 × 2 cm	Numerous small nodules (≤ 1.0 cm)	Brain, 5.1 × 3.6 cm
Symptoms	Dry cough	Numbness in the left upper limb	Abdominal pain, skin pimple	Weight loss, unable to walk
Hepatosplenomegaly	−	−	−	−
Hematologic abnormality^a^	−	−	−	−
*Morphology features*				
Growth pattern	Diffuse	Nodular	Diffuse	Diffuse
Histiocytes	Pleomorphic large cells, variable in size	Monomorphic large cells with epithelioid features	Monomorphic medium − sized cells	Monomorphic large cells with epithelioid features
Touton giant cells	+	+ , few	+	+ , few
Foamy cells	+	+	+ , many	+ , many
Emperipolesis	−	+ , few	−	−
Mitosis	Easily identified, 2 ~ 3/10 HPF	< 1/10 HPF	< 1/10 HPF	< 1/10 HPF
Interstitial fibrosis	−	−	+	−
Other cells in the stroma	SL, PC	SL, PC, EP	SL, PC, FB	SL, PC
*Immunohistochemical features*				
ALK1, Staining pattern	+ , Diffuse strong cytoplasmic; membranous	+ , Diffuse moderate cytoplasmic; membranous	+ , Diffuse moderate cytoplasmic	+ , Diffuse strong cytoplasmic
CD68	+	+	+	+
CD163	+	+	+	+
S − 100	−	+ , weak	−	−
CD1a	−	−	−	−
Langerin	−	−	−	−
P63	+ , p	ND	ND	ND
Ki-67	30%	1%	1%	5%
Others (negative)	CD20, CD3, CD30, CD45, CD79a, CK, CK8/18, CK7, CK-H, Desmon, EMA, Napsin A, PAX-5, PLAP, TTF1, SALL4, SMA, WT1	CD3, CD20, CD21, CD23, CD35, CD4, CD8, CK, EMA; GFAP, PR, SSTR2, TTF1,WT1	CD20, CD3, CD30, CK, Desmin, IgG4, SMA	CD20, CD3, CD30, EGFR, PLAP, SALL4, SSTR2, TdT
ALK-FISH^b^	+	+	+	+
Gene alteration	EML4-ALK(E19:A20) and TP53 mutation	KIF5B-ALK(K24:A20)	VRK2-ALK(V11:A20) and DCTN1-ALK(D26:A20) (liver biopsy specimen)	KIF5B-ALK(K24:A20)
Treatment	Chemoradiotherapy followed by inhibitor (alectinib and lorlatinib)	Surgical resection followed by inhibitor (alectinib)	Alectinib	Surgical resection followed by chemoradiotherapy and ALK inhibitor (crizotinib)
Follow-up	Stable disease for 24 months, and then progressive disease (2mo)	Alive with regressive disease (24 mo)	Alive with regressive disease (31 mo)	Died of disease (8 mo)

^a^including white blood cells, hemoglobin, platelet count, serum albumin level, and lactate dehydrogenase

^b^Break-apart FISH probe kit of ALK gene

*EP* Eosinophils; *F* Female; *FB* Fibroblasts; *LN* Lymph node; *M* Male; *mo* Month; *ND* Not done; *p* Partial positive; *PC* Plasma cells; *SL* Small lymphocytes; *y* Year; + , positive; − negative

### Case 1

#### Clinical presentation

**Fig. 1:**
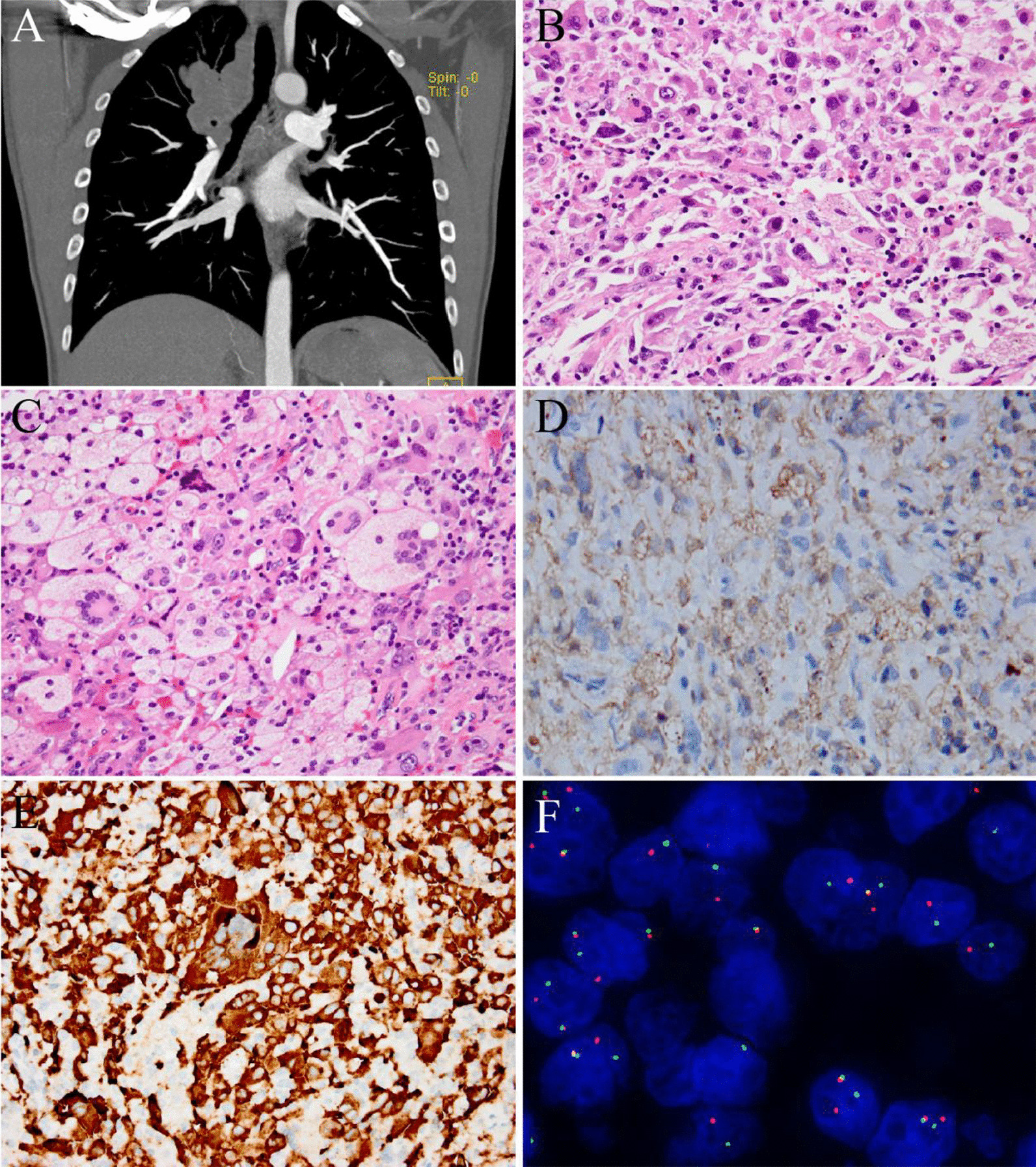
Radiological and pathological features of case 1. **A** Chest CT showed a large mass in the upper lobe of the right lung, involving the right anterior superior mediastinum, hilar, and hilar lymph nodes. **B, C** The mediastinal nodule exhibited large polymorphic histiocytes with frequent Touton giant cells. **D** The tumor cells were positive for CD68. **E** The tumor cells were positive for ALK1. **F** ALK-FISH (a break-apart probe) was positive. (Original magnification × 200 [B, C, D, E]; × 1000 [F])

A 38-year-old man presented with a nodular high-density shadow with a diameter of 0.9 cm in the upper lobe of the right lung during a routine health check-up, which was not treated at the time. Ten months later, he suffered from a dry cough. The examination of positron emission tomography-computed tomography (PET-CT) showed that the nodule had enlarged into an irregular, poorly demarcated mass (5.5 × 5 cm, SUV_max_ = 20.7) with multiple satellite lesions. The neoplasm involved the right anterior superior mediastinum, hilar lymph nodes, right superior lobe artery, and the superior vena cava (Fig. 1A). Fiber bronchoscopy revealed a cauliflower-like mass that blocked the bronchus of the upper-tip segment of the right lung. The clinical findings were initially suggestive of lung cancer; hence, mediastinal nodule biopsy and fiber bronchoscopy were performed.

#### Histological findings

Mediastinal nodule biopsy showed a diffuse growth pattern and was composed of large polymorphic histiocytes with abundant eosinophilic cytoplasm (Fig. 1B), which had round or ovoid, or lobulated nuclei. Touton giant and foamy cells were abundant (Fig. 1C). A few scattered giant heteromorphic cells with prominent eosinophilic nucleoli were observed. Mitosis was easily identified. No significant emperipolesis was observed. Fiber bronchoscopy biopsy showed a considerable pleomorphic histiocyte infiltration under the bronchial mucosal epithelium, similar to the mediastinal nodule lesion (see Additional file 1: Fig. S1A).

#### Immunophenotype and molecular analysis

The histiocytes were positive for CD68 (Fig. 1D), CD163 (see Additional file 1: Fig. S1B), and ALK1 (Fig. 1E), and negative for epithelial and lymphoid antibodies (Table 1). p63 was unevenly stained; the Ki-67 index was 30%; and FISH using a break-apart probe for ALK was positive (Fig. 1F). Next-generation sequencing revealed *EML4-ALK* gene fusion (see Additional file 1: Fig. S2A) and *TP53* gene mutations.

#### Treatment and follow-up

Before a definite diagnosis of ALK-positive histiocytosis, the patient was administered a “lung cancer” regimen [radiotherapy (21 times, total equivalent dose in 2 Gy/f (EQD2): mediastinum and lung 56.5 Gy, Hilar 71.9 Gy) combined with chemotherapy (paclitaxel + cisplatin + bevacizumab)]. After being diagnosed with ALK-positive histiocytosis, the patient received alectinib orally for 14 months. To prevent any possible resistance and to achieve better treatment effects, lorlatinib was used for eight months. During this period, CT examination showed that the lesion underwent no significant change. However, two months later, the patient developed bilateral pleural effusion, in which ALK-positive histiocytes were identified, indicating progressive disease. However, the patient refused further treatment, and was subsequently lost to follow-up.

### Case 2

#### Clinical presentation

**Fig. 2:**
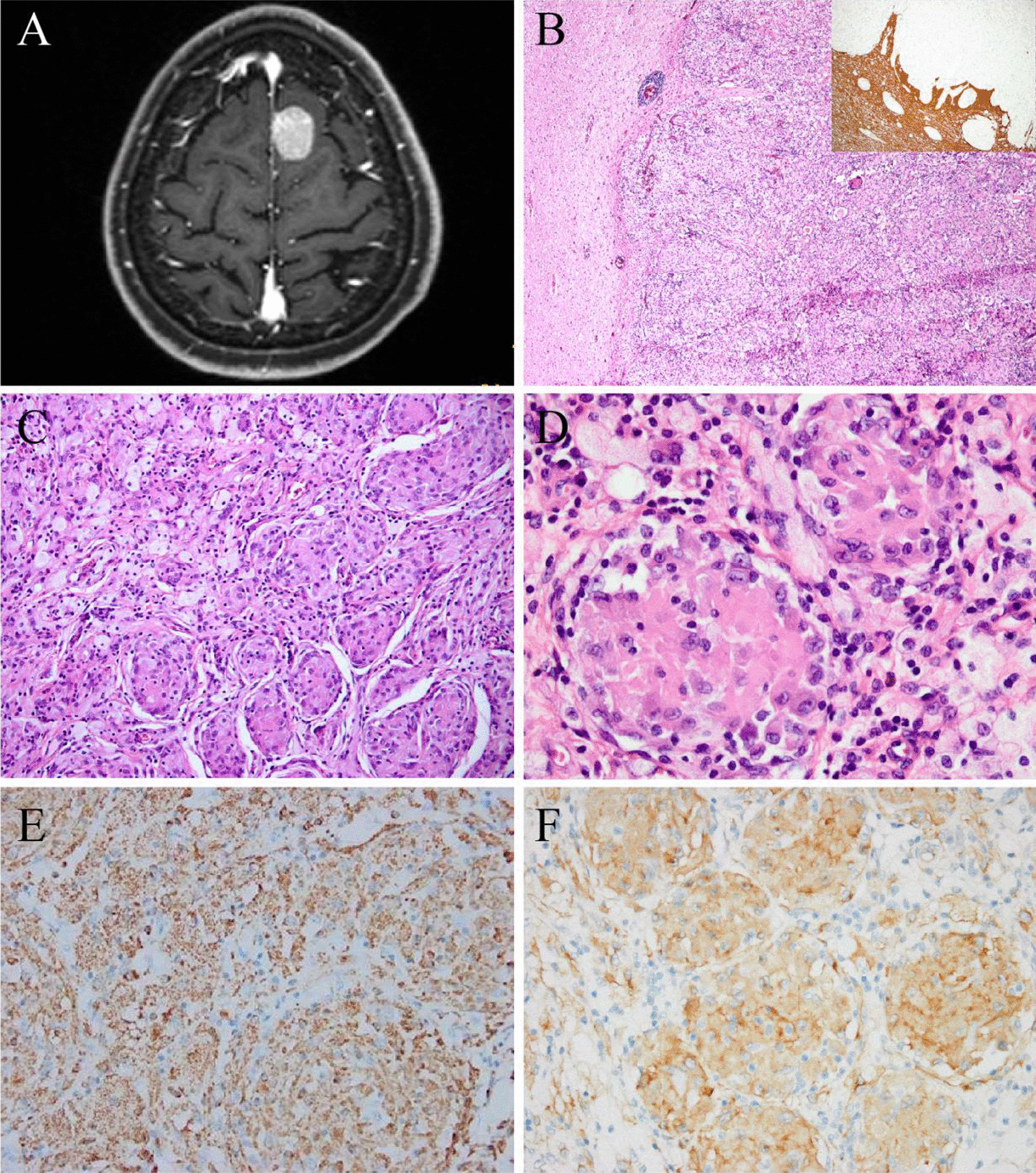
Radiological and pathological features of case 2. **A** CT showed a mass (2.5 × 2 cm in size) in the left frontal region near the cerebral falx of the brain. **B** The lesion infiltrated brain parenchyma, and GFAP staining showed an ill-defined lesion (inlet). **C** The lesion revealed a relatively uniform modular structure. **D** The tumor cells were large with round, ovoid, or clefted nuclei. **E** The tumor cells were positive for CD68. **F** The tumor cells were positive for ALK1. (Original magnification × 40 [B]; × 100 [C]; × 200 [E, F]; × 400 [D])

The patient was a 51-year-old woman with left upper limb numbness for > 20 days and a history of bilateral pulmonary nodules for four years. PET-CT showed that a well-demarcated mass of 2.5 × 2 cm in the left frontal region near the cerebral falx of the brain (Fig. 2A), numerous nodules in bilateral lungs (≤ 1.1 cm), and multiple enlarged lymph nodes in the mediastinum, bilateral hilum and abdominal cavity (≤ 1.2 cm). Imaging indicated meningioma and metastatic tumors.

#### Histological findings

The lesion infiltrated the brain parenchyma (Fig. 2B), where residual glial tissue could also be observed, although the boundary was clearly defined at low magnification and radiography. The lesion was diffusely distributed and formed a relatively uniform nodular pattern (Fig. 2C). The histiocytes were large with abundant eosinophilic cytoplasm, some clefted or lobulated nuclei, small nucleoli, and resembled meningothelial meningioma cells (Fig. 2D). Foamy cells and small lymphocytes were also observed. Scarce Touton giant cells and emperipolesis were seen.

#### Immunophenotype and molecular analysis

The histiocytes were positive for CD68 (Fig. 2E), CD163, ALK1 (Fig. 2F), and S100 (weak). The Ki-67 index was low (< 1%). Other markers were negative (Table 1). ALK-FISH was positive. *KIF5B-ALK* gene fusion was identified (see Additional file 1: Fig. S2B).

#### Treatment and follow-up

The patient underwent surgical resection of the mass, followed by oral alectinib maintenance therapy for 24 months. No recurrence of the intracranial tumor was observed. Bilateral pulmonary nodules and swollen lymph nodes were also significantly reduced.

### Case 3

#### Clinical presentation

**Fig. 3:**
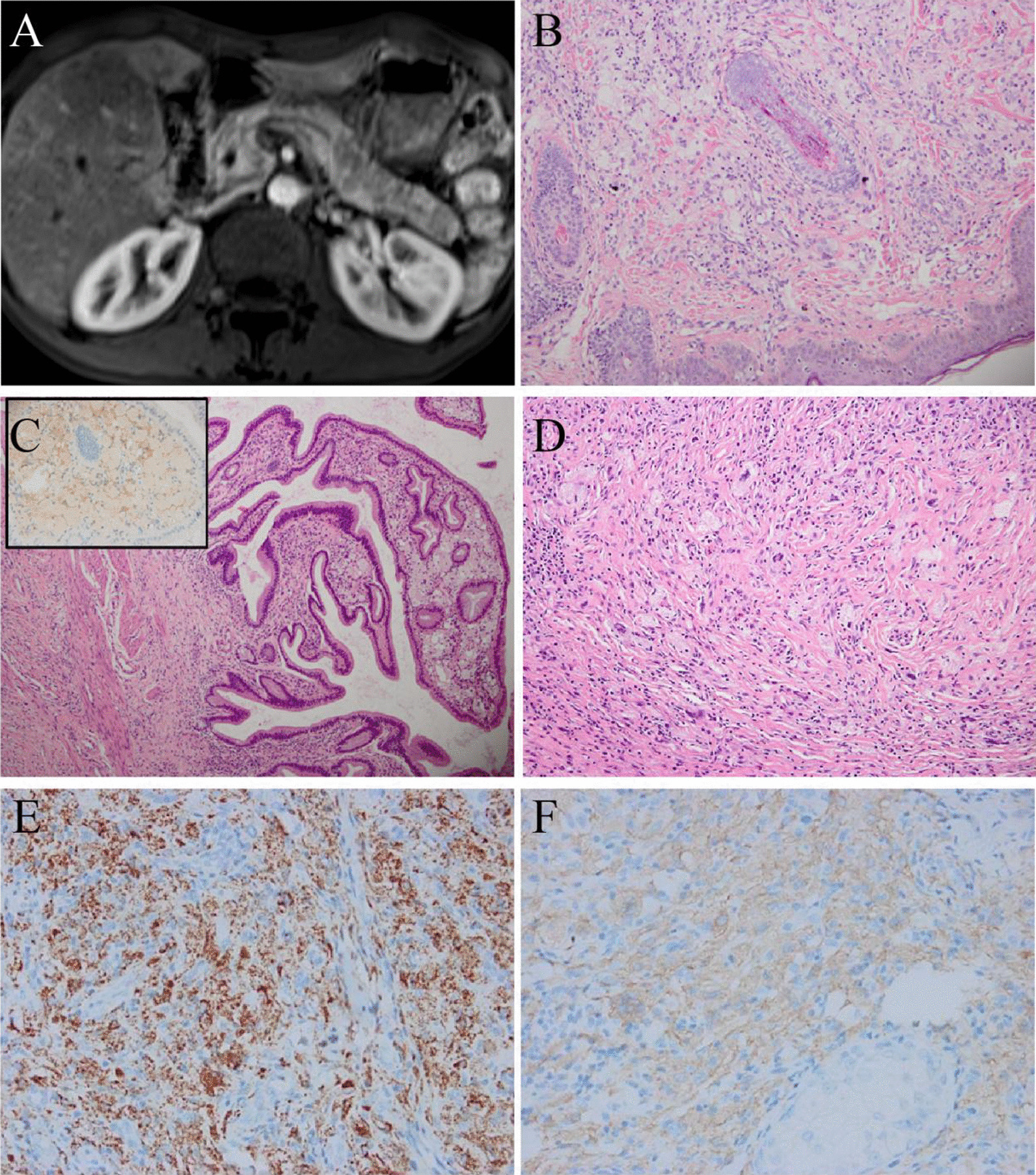
Radiological and pathological features of case 3. **A** MRI showed multiple patchy and/or nodular abnormal signal shadows in the liver. **B** The skin biopsy showed dermally infiltrates of histiocytes and Touton cells. **C** There were numerous foam cells with ALK-positive (inlet) in the mucosal layer of the gallbladder and fibrous tissue hyperplasia in the muscular layer. **D** The liver lesions showed prominent interstitial sclerosis, accompanied by foamy cells and Touton giant cells. **E** The histiocytes were positive for CD68 (Skin). **F** The histiocytes were positive for ALK1 (Skin). (Original magnification × 40 [C]; × 100 [B, D]; × 200 [E, F])

A 32-year-old man had abdominal pain for one year. Magnetic resonance imaging (MRI) revealed numerous nodules in the liver (Fig. 3A), pancreas, bilateral kidneys, an irregularly thickened gallbladder wall, and swollen lymph nodes in the abdominal cavity. Chest CT also revealed multiple small nodules (≤ 1.0 cm) in bilateral lungs. Additionally, numerous papules were observed on the face and trunk. The patient underwent a cholecystectomy and liver and facial skin biopsy.

#### Histological findings

The skin lesions showed many foam cells infiltrated in the dermis, accompanied by Touton giant cells (Fig. 3B). The lesions involved the entire thickness of the gallbladder wall (see Additional file 1: Fig. S1C). Numerous foam cells were observed in the mucous layer (Fig. 3C), muscular layer, and extraneous adipose tissue showed prominent interstitial sclerosis, accompanied by histocyte infiltration (mainly foamy cells and Touton giant cells) mixed with many lymphocytes and occasional plasma cells. Liver biopsy showed that the lesion was nodular and poorly demarcated from the surrounding liver tissue (see Additional file 1: Fig. S1D), and without hepatic sinusoid infiltration, histological features were similar to those of the gallbladder (Fig. 3D).

#### Immunophenotype and molecular analysis

Immunostaining revealed that all types of histiocytes were positive for CD68 (Fig. 3E), ALK (Fig. 3F), and CD163 (see Additional file 1: Fig. S1E), and negative for Langerin, CD1a, IgG4, SMA, desmin, and S-100. *ALK*-FISH was positive. *BRAF*-V600E was negative. *VRK2-ALK* and *DCTN1-ALK* were identified (see Additional file 1: Fig. S2C-D).

#### Treatment and follow-up

After 29 months of oral alectinib treatment, the papules on the face and trunk subsided, and the abdominal pain disappeared. The liver, pancreas, bilateral kidneys, and lung nodules were significantly reduced.

### Case 4

#### Clinical presentation

**Fig. 4:**
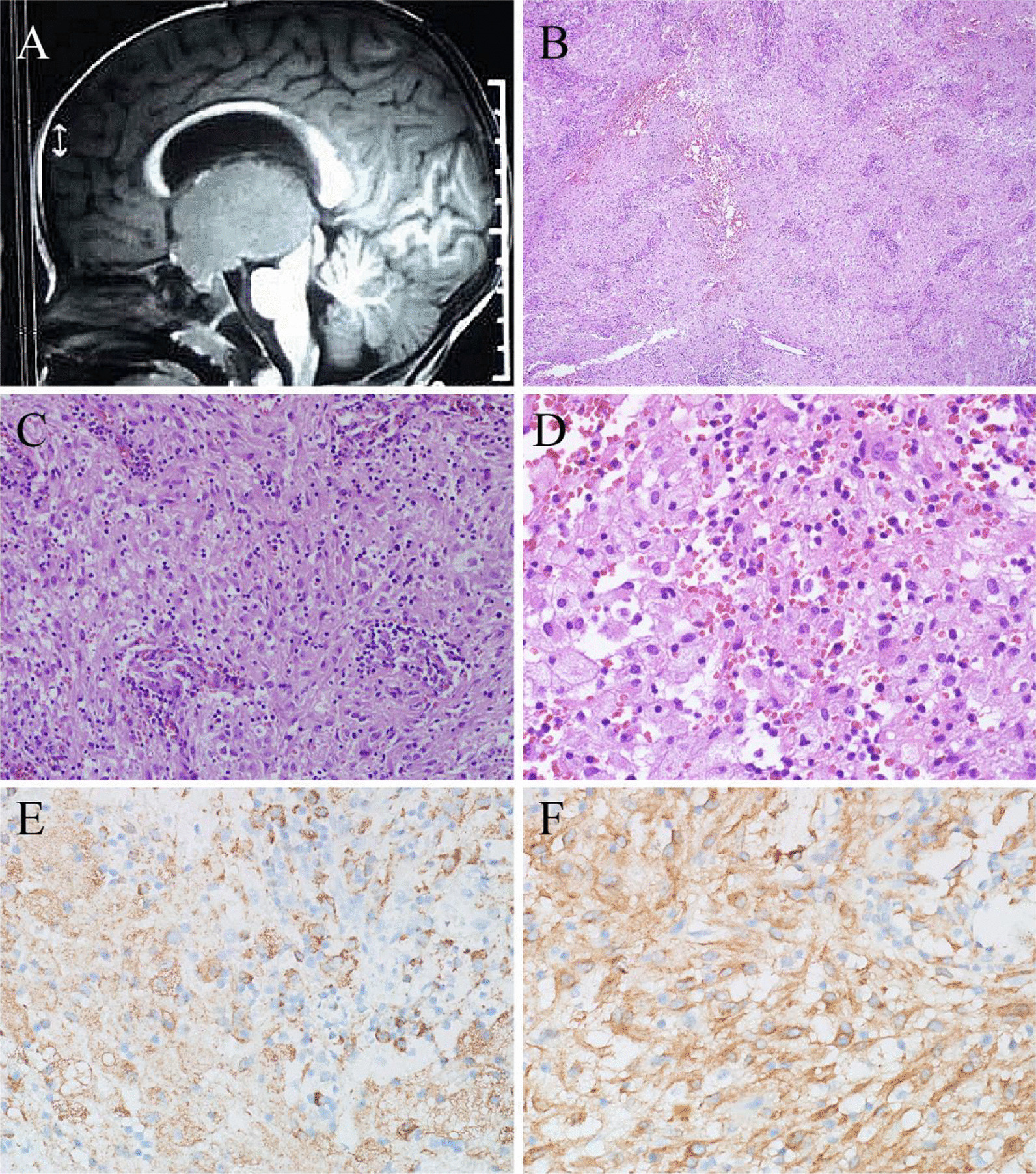
Radiological and pathological features of case 4. **A** The brain MRI showed a large mass (5.1 × 3.6 cm in size) in the third ventricle. **B** The lesion was diffusely distributed. **C** The histiocytes were epithelioid with abundant cytoplasm, accompanied by some lymphocytes. **D** Many foamy cells could be seen. **E** The histiocytes were positive for CD68. **F** The histiocytes were positive for ALK1. (Original magnification × 40 [B]; × 200 [C, E, F]; × 400 [D])

A 17-month-old boy was examined for weight loss (approximately 1 kg) and inability to walk. The brain MRI showed a large mass (5.1 × 3.6 cm) in the third ventricle (Fig. 4A) with slightly uneven density and significant dilation of bilateral ventricles. It was accompanied by interstitial brain edema; hence, a spermatocyte tumor was considered. Intraoperatively, the tumor boundary was unclear and could not be entirely removed. In addition, multiple nodules (≤ 1.2 cm) were also found in both lungs and liver. Several yellowish papules were observed on the skin of the chest wall (see Additional file 1: Fig. S1F) and right lower extremity (≤ 1.5 cm). Hemograms and marrow smears were normal. The patient had no hepatosplenomegaly, abnormal liver function, low fever, night sweats, nausea, or vomiting.

#### Histological findings

The histiocytes were diffusely distributed (Fig. 4B) and epithelioid (Fig. 4C), with abundant lightly stained cytoplasm, accompanied by many foamy cells and lymphocytes (Fig. 4D). Touton giant cells were rare.

#### Immunophenotype and molecular analysis

The epithelioid histiocytes, foamy cells, and Touton giant cells were all positive for CD68 (Fig. 4E) and CD163, ALK1 (Fig. 4F), and negative for CD20, CD3, CD30, CD1a, EGFR, Langerin, PLAP, SALL4, S-100, SSTR2, and TdT. *ALK*-FISH results were positive. A *KIF5B-ALK* gene fusion was identified (see Additional file 1: Fig. S2E).

#### Treatment and follow-up

The patient received chemotherapy (vincristine + prednisone acetate, followed by doxorubicin + vincristine + cyclophosphamide + prednisone acetate) combined with brain radiation and oral ALK inhibitors (crizotinib). The patient experienced an epileptic seizure during this period due to an intracranial infection (*Bacillus cereus*) and a high fever (39 °C). MRI also showed enlargement of the residual intracranial lesions. The lung and liver nodules showed no significant changes, and the skin papules partially subsided. Unfortunately, the disease progressed rapidly after the onset; the family declined further treatment and the patient died one month later.The overall survival of the patient was only eight months after surgery.

## Discussion

Since 2008, more than 60 cases of ALK-positive histiocytosis have been reported in the literature [1–19] (see detailed information in Additional file 2: Table S3–5). In 2018, Chang et al. classified ALK-positive histiocytosis into two types, systemic and localized, based on clinical manifestations [2]. Recently, an international retrospective study divided the entity into three groups: infants with multisystemic disease with liver and hematopoietic involvement (Group 1A), other patients with multisystemic disease (Group 1B), and patients with a single-system disease (Group 2) [19]. In the current study, the patients had no hepatosplenomegaly, hematological abnormalities, or infiltration of the hepatic sinusoids by large histiocytes. Therefore, we defined this group of diseases as multisystem ALK-positive histiocytosis without hematopoietic involvement, similar to "Group 1B", as previously described [19]. In addition, for case 1, it may be considered a single-system disease, given that it concerns one large pulmonary tumor with involvement of adjacent tissues and draining lymph nodes. This view is also acceptable.

Differential diagnosis of ALK-positive histiocytosis includes Erdheim-Chester disease (ECD), Langerhans cell histiocytosis (LCH), and Rosai-Dorfman disease (RDD). ECD is a clonal systemic proliferation of histiocytes, commonly having a foamy component and containing Touton giant cells. It mainly occurs in adults with a median age of 55–60 years and usually involves the diaphysis and metaphysis of the long bones of the limbs and is accompanied by pain in bones [20, 21]. The typical clinical and characteristic imaging manifestations, including a predominance of *BRAF-*V600E mutation, provide essential support for diagnosis. In contrast, ALK-positive histiocytosis is more common in infants and young adults and is mainly composed of non-foamy histiocytes with irregularly folded nuclei and ALK translocations. It does not involve the typical anatomic sites and lacks the *BRAF*-V600E mutation observed in ECD. LCH is a clonal neoplastic proliferation of Langerhans-type cells that expresses S100, CD1a, and Langerin and shows Birbeck granules on ultrastructural examination. The bones and adjacent soft tissues are the dominant sites of involvement in the solitary form. Approximately 50% of cases harbor *BRAF*-V600E mutations without ALK rearrangements [22, 23]. RDD may sometimes be confused with ALK-positive histiocytosis, but immunohistochemical staining and molecular detection can be used to differentiate between them. The latter is ALK-positive and has ALK gene rearrangement, whereas the former is the opposite.

In the current study, Case 1 was initially suspected of lung cancer. The neoplastic cells were positive for CD68 and CD163 and negative for epithelial antibodies; therefore, lung cancer was excluded. In addition, p63 was unevenly positive in this case; nevertheless, this was not specific to lung cancer and could be positive in many other tumors, including lymphoma [24–26], soft tissue tumors [27], germ cell tumors, and endocrine tumors [28]. In addition, the tumor cells were negative for CD30 and CD3, precluding the diagnosis of ALK-positive anaplastic large-cell lymphoma (ALCL). Moreover, mitosis was easily identified in the case, and whether this was related to the prognosis, still needs to be investigated further. In Case 2, the primary lesion was located in the left frontal region near the cerebral falx of the brain, and the histiocytes inside were epithelioid with a multinodular growth pattern. Therefore, a differential diagnosis of meningothelial meningioma was necessary. The histiocytes were positive for CD68, CD163, and ALK, and negative for EMA, PR, and SSTR2, which ruled out the diagnosis of meningioma. For Case 3, it was essential to differentiate it from IMT due to the many overlapping histologic features in both cases. First, the histiocytes were ALK1 positive, while the proliferating spindle cells in the stroma were negative for ALK1. However, histiocytes are ALK1-negative in IMT, spindle fibroblasts, and myofibroblasts, in approximately 50% of ALK-positive cases [29]. Second, the patient showed multiple organ involvement, which could be easily distinguished from IMT, which generally presents as an isolated mass. Case 4 exhibited an intracranial mass, pulmonary and liver nodules, skin papules, and mild anemia but no skeletal disease. Histologically, it was composed of epithelioid histiocytic cells and foamy cells, accompanied by the most common gene alteration of the *KIF5B-ALK* gene fusion in ALK-positive histiocytosis, and without *BRAF*-V600E, *TERT*, *IDH1*, *IDH2*, and *MGMT* gene mutations, which could be easily distinguished from ECD syndrome.

*KIF5B-ALK* has emerged as the most frequent molecular alteration in ALK-positive histiocytosis (83%, 44/53) (see Additional file 2: Table S3-5). Other less common partner genes of ALK fusion have also been reported, including *TPM3, COL1A2, TRIM33 CLTC, TFG-ALK, DCTN1-ALK,* and *EML4* [1, 2, 10, 16]. In the current study, two cases (Cases 2 and 4) had *KIF5B-ALK* gene fusion, and Case 3 harbored the *VRK2-ALK* and *DCTN1-ALK* fusion. *VRK2* is an atypical active Ser-Thr kinase involved in the control of cell cycle entry, apoptosis, and autophagy; it also affects signaling by mitogen activated protein kinases (MAPK) [30]. *DCTN1* is located on chromosome 2p13 and suspected to promote dimerization of *ALK* and subsequent kinase activation by transphosphorylation [31]. Moreover, *DCTN1-ALK* fusion has been reported in IMT [31, 32]. The *VRK2-ALK* and *DCTN1-ALK* fusion thus may lead to activation of *ALK* gene driving the tumorigenesis in the *ALK*-positive histiocytosis. For determining whether the gene fusions are related to the tumor’s biological behavior and the prognosis of patients, conducting further long-term follow-up observations and accumulating more case studies are necessary. Case 1 showed *EML4-ALK* fusion, which most commonly occurs in lung cancer, accounting for approximately 3–5% of all non-small cell lung cancers (NSCLC) [33]. However, the *EML4-ALK* fusion gene is not specific to NSCLC, which has also been observed in many other tumors, such as IMT [34], pediatric rhabdomyosarcoma [35], high-grade pediatric glioma [36], ovarian cancer [37], and cholangiocarcinoma [38]. Moreover, ALK-positive histiocytosis with *EML4-ALK* fusion in the lungs of a 52-year-old woman and a 17-year-old boy has also been reported [16, 19]. The above data indicate that ALK may have more partner genes in this disease, whether the different gene fusions are related to the tumor’s clinical characteristics and biological behavior is yet unknown. Searching for more cases for further research is recommended.

In our cohort, all patients were treated with ALK inhibitors. Two patients (Case 2 and 3) showed partial response after surgery. One patient (Case 1) presented stable disease for about two years and later showed progressive disease, which may be related to the combination of a large mass, *TP53* gene mutation, and a high index of Ki-67. Case 4 presented with a poor response to ALK inhibitors and chemotherapy and died of the disease in a short time. In an international retrospective study [19], it was observed that 11 patients treated with ALK inhibitors sustained objective responses. In addition, other studies have reported favorable outcomes of ALK inhibitors with ALK-positive histiocytosis [2, 5, 12, 17, 19]. However, these are all retrospective studies, and it remains controversial whether ALK inhibitor should be used as a first- or second-line treatment or when the disease is refractory to conventional therapies. Moreover, there is no consensus on the optimal treatment regimen and duration of ALK inhibitors, and long-term prognosis requires further observation. In addition, one particular case has been described, in which bone marrow biopsy showed concomitant chronic lymphocytic leukemia/small lymphocytic lymphoma and ALK-positive histiocytosis [11]. Four years after ibrutinib therapy, the patient remained free of both diseases, suggesting that BTK inhibitors may be effective in treating histiocytic diseases.

Involvement of the central nervous system (CNS) has become the most common manifestation of ALK-positive histiocytosis [19]. We identified 19 cases of ALK-positive histiocytosis with CNS involvement in the literature [2, 5, 9, 12, 17, 19]. The clinicopathological features of these cases and the two cases in our study are summarized in Table 2. Twenty-one cases (including our cohort and cases from the literature) were collected, including 11 multisystem cases without hematopoietic involvement and 10 single-system (CNS) cases. Cases were more common in women than men (2.5:1), and the majority were in children and adolescents (81%, 17/21), with a median age of 7 years. *KIF5B* was identified in all patients (100%, 19/19). In single-system lesions with only CNS involvement, 78% of patients (7/9) were cured by surgery and/or chemotherapy and ALK inhibitor, while in patients with multisystem involvement, the cure rate was only 27% (3/11), and two young children died of the disease in a short time. This suggests that the prognosis of patients with multisystem lesions with CNS involvement is worse than that of patients with single-system lesions (only CNS involvement), especially in young children.

**Table 2:** ALK-positive histiocytosis with CNS involvement reported in the literature

Authors	Gender	Age	Orangs involved	Gene fusion	Treatment	Follow-up
1. Chang, et al. [2]	M	2 y 9 mo	CNS, Intestine, hematopoietic system	ALK-FISH +	Steroids and chemotherapy (ECICM)	DOD (2 mo)
2. Qiu et al. [12]	M	49 y	CNS, bone, soft tissue, visceral organs, pleura	KIF5B-ALK	Gamma knife, chemotherapy, and ALK inhibitor	SD (7 mo)
3. Kashima et al. [5]	F	16 y	CNS, breast, pancreas, and lungs	KIF5B-ALK	Surgical resection followed by inhibitor	NED (44 mo)
4. Rossi et al. [17, 19]	M	10 mo	CNS, lung, liver, soft tissue	KIF5B-ALK	Surgical resection followed by chemotherapy (vinblastine/prednisone), combined with ALK inhibitor	NED (13 mo)
Kemps et al. [19]						
5.	F	2 y	CNS, bone, lung, liver, skin, soft tissue, kidney, breast, pancreas, LN	KIF5B-ALK	Chemotherapy (vinblastine/prednisone) followed by ALK inhibitor	ARD (21 mo)
6.	F	10 y	CNS, bone, lung, cervix, thyroid, submandibular salivary gland, LN	KIF5B-ALK	ALK inhibitor	NED (2 y)
7.	F	19 y	CNS/PNS, bone, lung, liver, breast, pancreas, LN	KIF5B-ALK	Chemotherapy (cladribine), followed by ALK inhibitor	ARD (2 y)
8.	F	28 y	CNS/PNS, bone	KIF5B-ALK	ALK inhibitor	ARD (9 mo)
9.	F	41 y	CNS, bone, lung, skin, soft tissue, LN	KIF5B-ALK	Interferon-a followed by ALK inhibitor	ARD (6 y)
Lucas et al. [9]						
10.	F	7 y	CNS	KIF5B-ALK	Surgical resection	NED (1 y)
11.	F	10 y	CNS	KIF5B-ALK	Surgical resection	NED (6 mo)
12. Rossi et al. [17, 19]	F	11 y	CNS	KIF5B-ALK	Surgical resection	NED (9 mo)
Kemps et al. [19]						
13.	F	7 mo	CNS	KIF5B-ALK	Corticosteroids, followed by ALK inhibitor	ARD (5 mo)
14.	F	9 mo	CNS	ALK-FISH +	N/A	LTF
15.	M	2.5 y	CNS	KIF5B-ALK	Chemotherapy followed by ALK inhibitor	NED (16 mo)
16.	F	3 y	CNS	KIF5B-ALK	Corticosteroids, followed by surgical resection	NED (12 mo)
17.	F	7 y	CNS	KIF5B-ALK	Chemotherapy (clofarabine)	NED (6 mo)
18.	F	13 y	CNS/PNS	KIF5B-ALK	Surgical resection followed by ALK inhibitor	ARD (2.5 y)
19.	M	20 y	CNS	KIF5B-ALK	Surgical resection	NED (10 mo)
Current						
20.	F	51 y	CNS, lung, LN	KIF5B-ALK	Surgical resection followed by ALK inhibitor	ARD (24 mo)
21.	M	17 mo	Brain, lung, liver, skin	KIF5B-ALK	Surgical resection followed by chemoradiotherapy and ALK inhibitor	DOD (8 mo)

*ARD* Alive with regressive disease; *CNS* Central nervous system; *DOD* Died of disease; *ECICM* Etoposide, cyclosporine, immunoglobulins, cytarabine, methotrexate; *F* Female; *LN* Lymph node; *LTF* Lost to follow-up; *M* Male; *mo*, Month; *NED* No evidence of disease; *PNS* Peripheral nervous system; *SD* Stable disease; *y* Year

## Conclusions

In conclusion, four cases of multisystem ALK-positive histiocytosis without hematopoietic involvement are reported. All cases showed unique clinical features, variable histological features, and robust and durable responses to ALK inhibitors in adult patients but a poor response in young children with CNS involvement. Histological lesions and neoplasms should be assessed for ALK immunostaining, particularly in cases of multiorgan involvement and in those instances where the lesions are not amenable to resection. In addition, it is still necessary to accrue more cases of the disease for further observation and study.

## Methods

### Case selection

Data of four cases of ALK-positive histiocytosis were reviewed between 2019 and 2021 from the database of the Department of Pathology, West China Hospital, Sichuan University. Detailed clinical data, including age, sex, symptoms, clinical course, imaging findings, laboratory examinations, and treatment options, were collected from electronic medical records. Follow-up data were obtained via telephone interviews and/or medical records.

### Immunohistochemistry analyses

Tissue sections of 4 µm thickness cut from 10% formalin-fixed, paraffin-embedded tissue blocks were used for immunostaining (Dako Autostainer Link 48). The antibodies used were as follows: ALK (ALK1, Dako), CD68 (PGM1, Maixin, China), CD163 (10D6, Maixin), CD1a (EP80, Zhongshan, China), Langrin (12D6, Zhongshan), S-100 (4C4.9 Zhongshan), and CD30 (Ber-H2, Maixin). Others are listed in Additional file 2: Table S1. Appropriate positive and negative controls were used in this study.

### Fluorescence in-situ hybridization

The *ALK* break-apart FISH Probe Kit (Vysis, Downers Grove, USA) was used to detect gene translocation using fluorescence in-situ hybridization (FISH), as previously described [2]. Fifty non-overlapping tumor cell nuclei were scored for interpretation of each case. *ALK* gene translocation was indicated by a split signal, defined as the separation of signals by two or more signal diameters. The result was considered positive if more than 15% of the assessed nuclei had split signals.

### Next-generation sequencing assay

A commercially available capture-based targeted sequencing panel (solid tumors 56 gene detection kit, Burning Rock Biotech Ltd., Guangzhou, China.) was used to identify the partner gene fused to *ALK* (see Additional file 2: Table S2). DNA was hybridized with capture probe baits and selected with a magnetic bead. A bioanalyzer high-sensitivity DNA assay was used to assess quality and size range. The samples were then sequenced on an Illumina MiseqDx platform with paired-end reads.

## Supplementary Information

**Additional file 1: Fig. S1.** Clinicopathologic features of ALK-positive histiocytosis. A, Case 1-the fiber bronchoscopy biopsy exhibited large histiocytes with frequent Touton giant cells similar to the mediastinal lesion. B, Case 1-the tumor cells were positive for CD163 (mediastinal lesion). C, Case 3-The lesions involved the entire thickness of the gallbladder wall. D, Case 3-the liver lesions were nodular and poorly demarcated. E, Case 3-the histiocytes were positive for CD163 (skin). F, Case 4-a yellowish papula could be seen on the skin of the chest wall. (Original magnification ×40 [C,D]; ×200 [A,B,E]).** Fig. S2.** Molecular features of ALK-positive histiocytosis. A, Case 1- EML4-ALK gene fusion with breakpoints at EML4 exon 19 and ALK exon 20. B, Case 2 -KIF5B-ALK gene fusion with breakpoints at KIF5B exon 24 and ALK exon 20. C, Case 3 - VRK2-ALK gene fusion with breakpoints at VRK2 exon 11 and ALK exon 20. D, Case 3 -DCTN1-ALK gene fusion with breakpoints involving DCTN1 exon 26 and ALK exon 20. E, Case 4 -KIF5B-ALK gene fusion with breakpoints at KIF5B exon 24 and ALK exon 20.

**Additional file 2: Table S1.** Other antibodies and special stain used in this study.** Table S2.** The gene panel used in this study (n=56).** Table S3.** Multisystem ALK-positive histiocytosis with hematopoietic involvement reported in the literature.** Table S4.** Multisystem ALK-positive histiocytosis without hematopoietic involvement reported in the literature. Table S5. Single-system of ALK-positive histiocytosis reported in the literature

## Data Availability

All data generated or analyzed during the current study are included in this published article.

## Abbreviations

ALK: Anaplastic lymphoma kinase
IMT: Inflammatory myofibroblastic tumors
FISH: Fluorescence in-situ hybridization
PET-CT: Positron emission tomography-computed tomography
CT: Computed tomography
MRI: Magnetic resonance imaging
ECD: Erdheim-Chester disease
LCH: Langerhans cell histiocytosis
RDD: Rosai-Dorfman disease
NSCLC: Non-small cell lung cancers
CNS: Central nervous system
