# Supplementary material for: Multisystem ALK-positive histiocytosis: a multi-case study and literature review
Source: Orphanet J Rare Dis. 2023 Mar 13;18:53. doi: 10.1186/s13023-023-02649-x (PMC10010018; doi:10.1186/s13023-023-02649-x)
Supplement: Supplementary file 1 — Additional file 1: Fig. S1. Clinicopathologic features of ALK-positive histiocytosis. A, Case 1-the fiber bronchoscopy biopsy exhibited large histiocytes with frequent Touton giant cells similar to the mediastinal lesion. B, Case 1-the tumor cells were positive for CD163 (mediastinal lesion). C, Case 3-The lesions involved the entire thickness of the gallbladder wall. D, Case 3-the liver lesions were nodular and poorly demarcated. E, Case 3-the histiocytes were positive for CD163 (skin). F, Case 4-a yellowish papula could be seen on the skin of the chest wall. (Original magnification ×40 [C,D]; ×200 [A,B,E]). Fig. S2. Molecular features of ALK-positive histiocytosis. A, Case 1- EML4-ALK gene fusion with breakpoints at EML4 exon 19 and ALK exon 20. B, Case 2 -KIF5B-ALK gene fusion with breakpoints at KIF5B exon 24 and ALK exon 20. C, Case 3 - VRK2-ALK gene fusion with breakpoints at VRK2 exon 11 and ALK exon 20. D, Case 3 -DCTN1-ALK gene fusion with breakpoints involving DCTN1 exon 26 and ALK exon 20. E, Case 4 -KIF5B-ALK gene fusion with breakpoints at KIF5B exon 24 and ALK exon 20. [file 13023_2023_2649_MOESM1_ESM.docx]

| 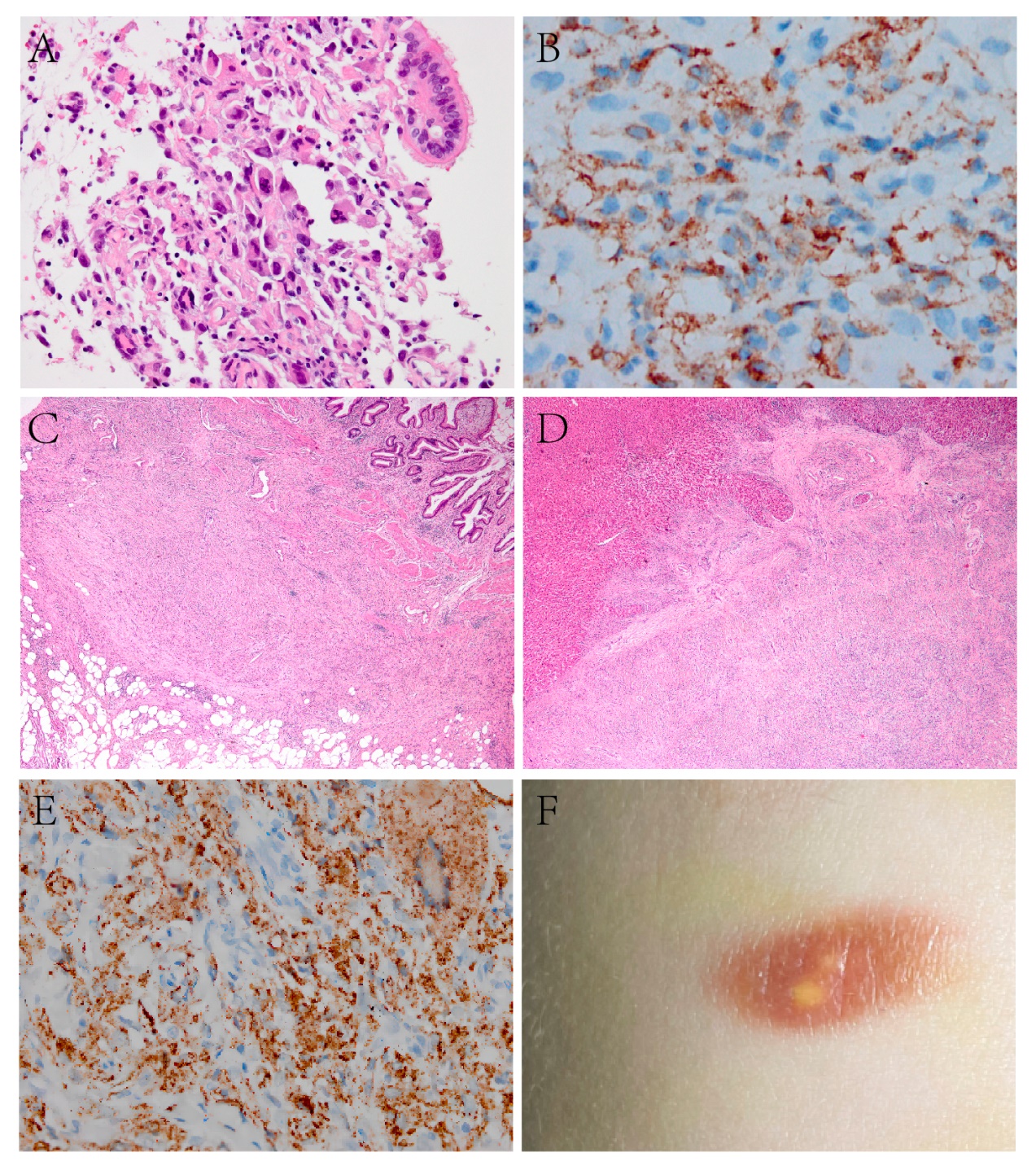 |
| --- |
| **Figure S1**. Clinicopathologic features of ALK-positive histiocytosis. A, Case 1-the fiber bronchoscopy biopsy exhibited large histiocytes with frequent Touton giant cells similar to the mediastinal lesion. B, Case 1-the tumor cells were positive for CD163 (mediastinal lesion). C, Case 3-The lesions involved the entire thickness of the gallbladder wall. D, Case 3-the liver lesions were nodular and poorly demarcated. E, Case 3-the histiocytes were positive for CD163 (skin). F, Case 4-a yellowish papula could be seen on the skin of the chest wall. (Original magnification ×40 [C,D]; ×200 [A,B,E]). |

| 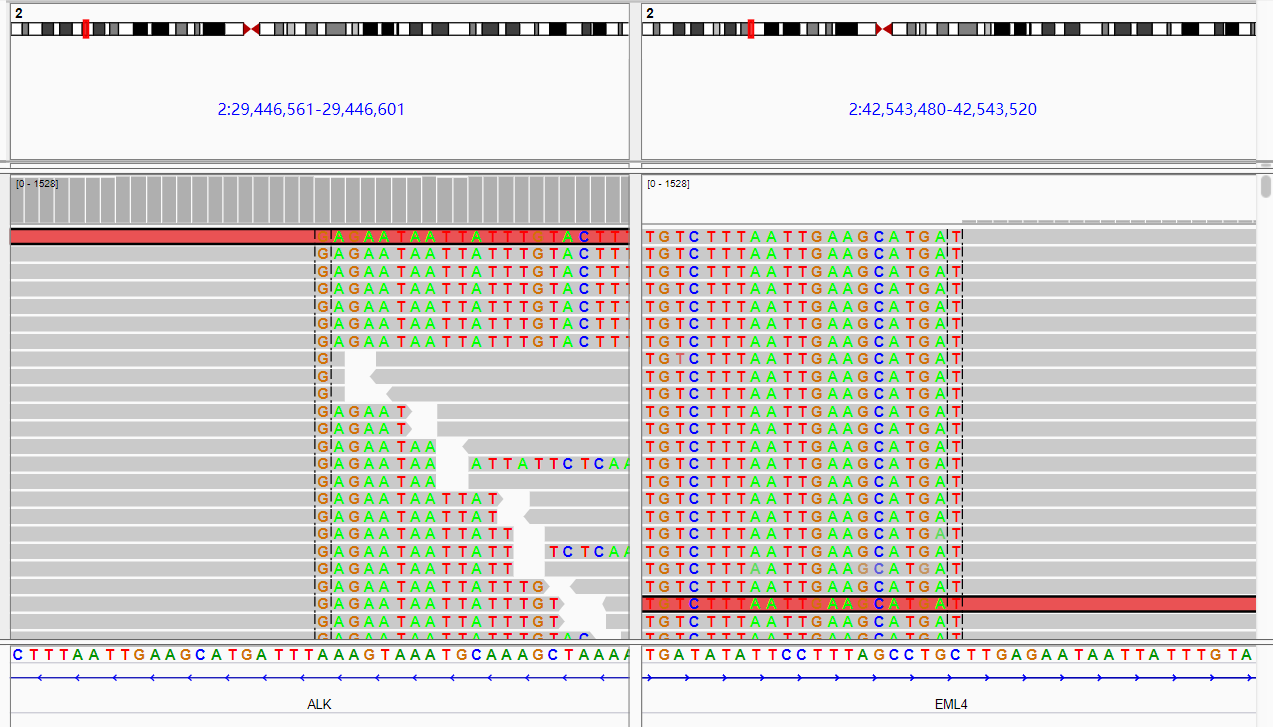  **C**  **A** | 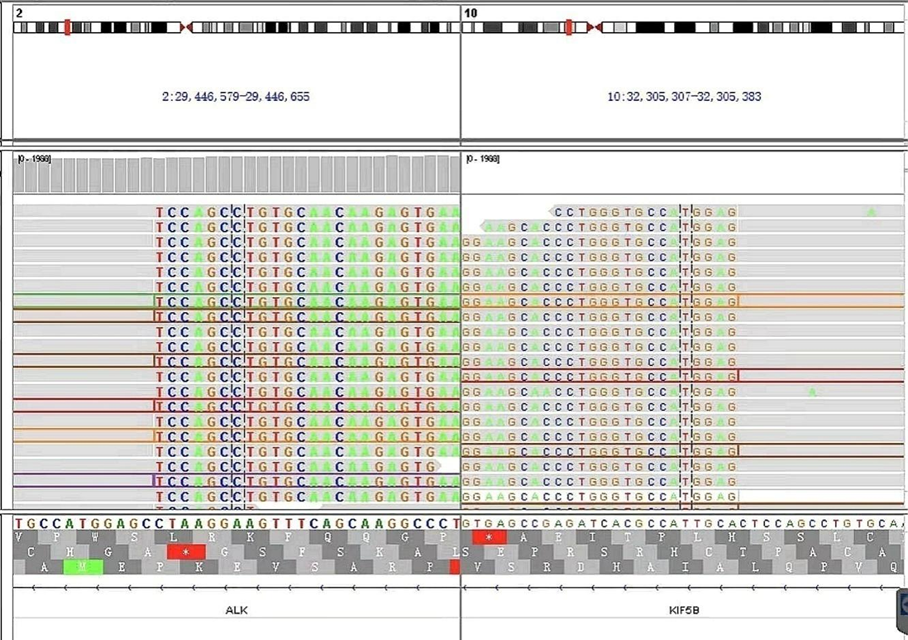  **D**  **B** |
| --- | --- |
| 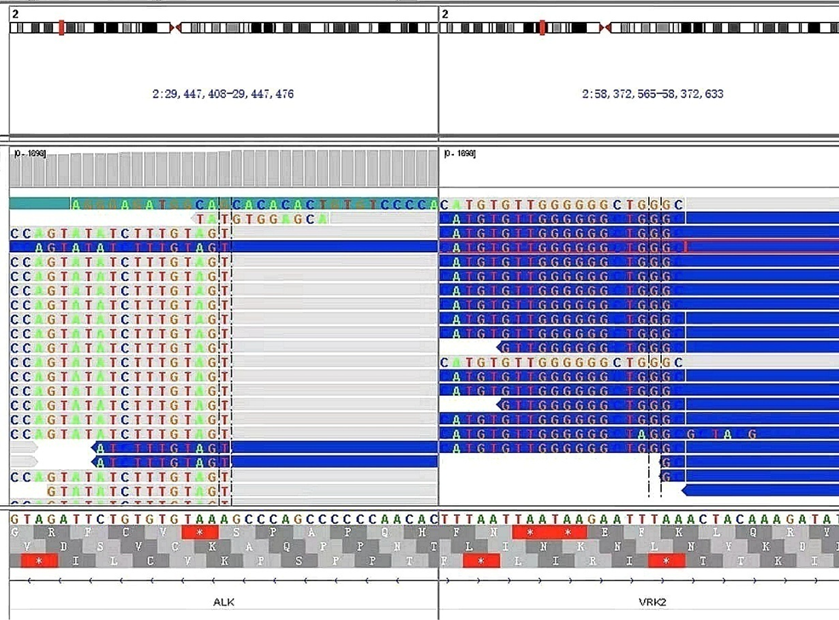 | 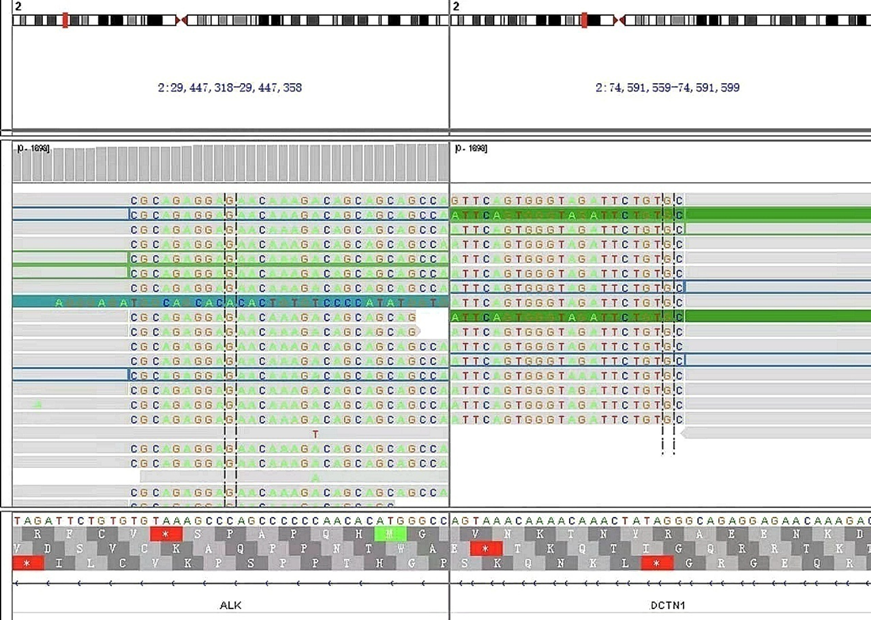 |
| 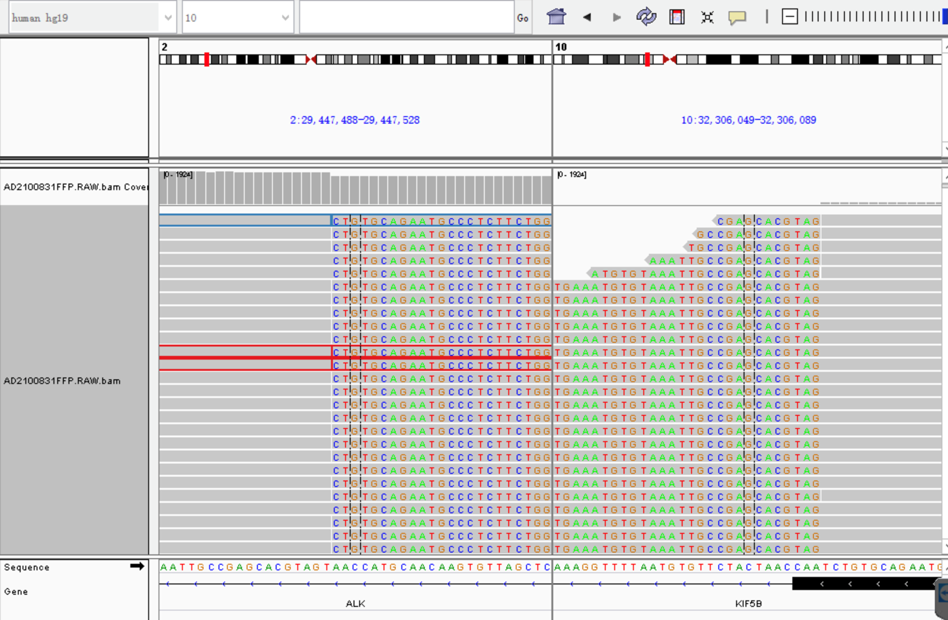  **E** |  |
| **Figure S2.** Molecular features of ALK-positive histiocytosis. A, Case 1- EML4-ALK gene fusion with breakpoints at EML4 exon 19 and ALK exon 20. B, Case 2 -KIF5B-ALK gene fusion with breakpoints at KIF5B exon 24 and ALK exon 20. C, Case 3 - VRK2-ALK gene fusion with breakpoints at VRK2 exon 11 and ALK exon 20. D, Case 3 -DCTN1-ALK gene fusion with breakpoints involving DCTN1 exon 26 and ALK exon 20. E, Case 4 -KIF5B-ALK gene fusion with breakpoints at KIF5B exon 24 and ALK exon 20. | |
