# Supplementary material for: Multisystem ALK-positive histiocytosis: a multi-case study and literature review
Source: Orphanet J Rare Dis. 2023 Mar 13;18:53. doi: 10.1186/s13023-023-02649-x (PMC10010018; doi:10.1186/s13023-023-02649-x)
Supplement: Supplementary file 2 — Additional file 2: Table S1. Other antibodies and special stain used in this study. Table S2. The gene panel used in this study (n=56). Table S3. Multisystem ALK-positive histiocytosis with hematopoietic involvement reported in the literature. Table S4. Multisystem ALK-positive histiocytosis without hematopoietic involvement reported in the literature. Table S5. Single-system of ALK-positive histiocytosis reported in the literature [file 13023_2023_2649_MOESM2_ESM.docx]

**Table S1**. **Other antibodies and special stain used in this study**

| **Antibody** | **Clone** | **Manufacturer** |
| --- | --- | --- |
| CD20 | L26 | Dako |
| CD21 | EP3093 | Maixin |
| CD23 | SP23 | Maixin |
| CD3ε | Polyclonal | Dako |
| CD30 | Ber-H2 | Maixin |
| CD35 | EP197 | Maixin |
| CD4 | SP35 | Maixin |
| CD45 | Kit-0024 | Maixin |
| CD8 | C8/144B | Maixin |
| CD56 | 123C3 | Zhongshan |
| CD79a | SP18 | Maixin |
| CK | AE1/AE3 | Zhongshan |
| CK-H | 34βE12 | Maixin |
| CK7 | MX053 | Maixin |
| CK8/18 | 5D3 | Maixin |
| Desmin | EP15 | Zhongshan |
| EGFR | OTI3H2 | Zhongshan |
| EMA | E29 | Zhongshan |
| GFAP | EP13 | Zhongshan |
| IgG4 | EP138 | Zhongshan |
| Ki-67 | MIB-1 | Dako |
| Langerin | 12D6 | Zhongshan |
| Napsin A | MX015 | Maixin |
| P16 | 6H12 | Maixin |
| PAX-5 | ZP007 | Zhongshan |
| PR | 1E2 | Ventan |
| PLAP | EP194 | Zhongshan |
| SALL4 | 6E3 | Zhongshan |
| SMA | UMAB237 | Zhongshan |
| SSTR2 | EP149 | Zhongshan |
| TdT | SEN28 | Zhongshan |
| TTF-1 | MAB-0677 | Maixin |
| WT1 | WT49 | Maixin |
| Periodic Acid-Schiff stain | MST-8050/8051 | Maixin |
| acid-fast stain | MST-8009/8010 | Maixin |

**Table S2**. The gene panel used in this study (n=56)

| AKT1 | **ALK** | ARAF | ATM | BIM | **BRAF** | BRCA1 | BRCA2 | CCND1 | CDK4 |
| --- | --- | --- | --- | --- | --- | --- | --- | --- | --- |
| CDK6 | CDKN2A | CTNNB1 | CYP2D6 | DDR2 | DPYD | EGFR | ERBB2 | ERBB3 | ERBB4 |
| FGF19 | FGF3 | FGF4 | FGFR1 | FGFR2 | FGFR3 | FLT3 | HRAS | JAK1 | JAK2 |
| KDR | KIT | KRAS | MAP2K1 | MET | MTOR | MYC | NRAS | NRG1 | NTRK1 |
| NTRK2 | NTRK3 | PDGFRA | PIK3CA | PTCH1 | PTEN | RAF1 | RB1 | RET | ROS1 |
| SMO | STK11 | **TP53** | TSC1 | TSC2 | UGT1A1 |  |  |  |  |

**Table S3：Multisystem ALK-positive histiocytosis with hematopoietic involvement reported in the literature.**

| **Authors** | **Gender** | **Age** | **Orangs involved** | **Gene fusion** | **Treatment** | **Follow-up** |
| --- | --- | --- | --- | --- | --- | --- |
| Chan,et al^[1]^(2008) |  |  |  |  |  |  |
| 1 | F | Neonate | Spleen, liver, skin, HS | TPM3-ALK | Chemotherapy (dexamethasone and etoposide) | NED at 12 y |
| 2 | F | 3 mo | Spleen, liver, HS | ALK-positive by IHC | Chemotherapy (dexamethasone and etoposide) | NED at 14 y |
| 3 | F | 3 mo | Spleen, liver, HS | ALK-positive by IHC | Antibiotics | NED at 13 y |
| Chang,et al^[2]^(2019) |  |  |  |  |  |  |
| 4 | F | 2 mo | Spleen, liver, HS | KIF5B-ALK | Chemotherapy(vinblastine and etoposide) | NED at 2 y |
| 5 | M | 3 mo | Liver, skin, Kidney, lung, HS | KIF5B-ALK | Chemotherapy (ALCL99 protocol) | NED at 4 y |
| 6 | M | 2 y  9 mo | CNS, intestine, HS | ALK-FISH+ | Steroids and chemotherapy (ECICM) | DOD (2 mo) |
| Huang,et al^[8,20]^(2018) |  |  |  |  |  |  |
| 7 | F | 27 d | Liver, Kidney, spleen, HS | ALK-FISH+ | Chemotherapy (prednisone and vinblastine) | NED (7 y) |
| 8 | M | 2 d | Liver, HS | ALK-positive by IHC | N/A | LTF |
| Kemps, et al^[20]^ (2022) |  |  |  |  |  |  |
| 9 | F | 0 d | Liver, spleen, lung, possibly Kidney, HS | N/A | Intravenous immunoglobulin, corticosteroids and supportive care | NED (3.5 y) |
| 10 | M | 1 mo | Liver, spleen, Kidney, skin, HS | KIF5B-ALK | Chemotherapy (vinblastine/ dexamethasone/ methotrexate) | DOD (1 mo) |
| 11 | F | 2 mo | Liver, spleen, HS | N/A | Supportive care | NED (4.5 y) |
| 12 | F | 4 mo | Liver, HS | ALK-FISH+ | N/A | LTF |
| 13 | M | 5 mo | Liver, spleen, HS | CLTC-ALK | Corticosteroids followed by chemotherapy | NED (4 y) |

CNS, central nervous system; d, day; DOD, died of disease; ECICM, etoposide, cyclosporine, immunoglobulins, cytarabin, methotrexate; F, female; HS, hematopoietic system; LTF, lost to follow-up; M, male; mo, month; N//A, not available; NED, no evidence of disease; y, year.

**Table S4：Multisystem ALK-positive histiocytosis without hematopoietic involvement reported in the literature.**

| **Authors** | **Gender** | **Age** | **Organ involved** | **Gene fusion** | **Treatment** | **Follow-up** |
| --- | --- | --- | --- | --- | --- | --- |
| 1 Ross,et al^[12 ]^(2017) | M | 40 y | Bone, liver, soft tissue | KIF5B-ALK | Chemoradiotherapy and ALK inhibition | ARD (9 mo) |
| 2 [Syrykh,](javascript:void(0);)et al^[11]^*(2021) | F | 37 y | BM, breast | ALK -FISH+ | BTK inhibitors (ibrutinib) | NED (4 y) |
| 3 Qiu,etal^[ 12 ]^(2021) | M | 49 y | CNS, bone, soft tissue,  visceral organs, pleura | KIF5B-ALK | Gamma knife, chemotherapy，and ALK inhibition | SD (7 mo) |
| 4 Kashima,et al^[5]^(2021) | F | 16 y | CNS,breast; pancreas,and lungs. | KIF5B-ALK | Surgical resection followed by ALK inhibition | NED (44 mo) |
| 5 Rossi,et al^[17，20]^(2021) | M | 10 mo | CNS, lung, liver, soft tissue | KIF5B-ALK | Surgical resection followed by chemotherapy (vinblastine/prednisone), combined with ALK inhibition | NED (13 mo) |
| Kemps, et al^[20]^ (2022) |  |  |  |  |  |  |
| 6 | F | 3 mo | Bone, lung, liver | TPM3-ALK | Chemotherapy (vinblastine/prednisone) | NED (2 y) |
| 7 | F | 9 mo | Lung, skin, Kidney | ALK-FISH+ | Chemotherapy (vinblastine/prednisone) | NED (2 y) |
| 8 | F | 2 y | CNS, bone, lung, liver, skin, soft tissue,kidney, breast, pancreas, LN | KIF5B-ALK | Chemotherapy (vinblastine/prednisone) followed by ALK inhibition | ARD (21 mo) |
| 9 | F | 10 y | CNS, bone, lung,cervix, thyroid, submandibular salivary gland, LN | KIF5B-ALK | ALK inhibition | NED (2 y) |
| 10 | F | 19 y | CNS/PNS, bone, lung, liver, breast, pancreas,LN | KIF5B-ALK | Chemotherapy (cladribine) followed by ALK inhibition | ARD (2 y) |
| 11 | M | 21 y | Liver, skin, colorectum | TFG-ALK | Corticosteroids | DOD (2 mo) |
| 12 | F | 28 y | CNS/PNS, bone | KIF5B-ALK | ALK inhibition | ARD (9 mo) |
| 13 | M | 29 y | PNS | KIF5B-ALK | Corticosteroids, followed by pegylated interferon-a | NED (2.5 y) |
| 14 | F | 41 y | CNS, bone, lung, skin, soft tissue,LN | KIF5B-ALK | Interferon-a followed by ALK inhibition | ARD (6 y) |

*The case had a history of CLL/SLL. A bone marrow biopsy showed concomitant CLL/SLL and ALK-positive histiocytosis.

ARD, alive with regressive disease; CNS, central nervous system; DOD, died of disease; F, female; M, male; mo, month; LN, lymph node; NED, no evidence of disease; PNS, peripheral nervous system; SD, stable disease; y, year.

**Table S5：Single-system of ALK-positive histiocytosis reported in the literature .**

| **Authors** | **gender** | **Age** | **Organ involved** | **Gene fusion** | **Treatment** | **Follow-up** |
| --- | --- | --- | --- | --- | --- | --- |
| Chang,et al^[2]^(2019) |  |  |  |  |  |  |
| 1 | M | 2y 3mo | Nasal skin papule | KIF5B-ALK | Surgical resection | NED(2.5y ) |
| 2 | M | 15 y | Cavernous sinus | KIF5B-ALK | ALK inhibition | NED (6 mo ) |
| 3 | M | 16 y | Skin/soft tissue | COL1A2-ALK | Surgical resection | NED (3 y ) |
| 4 | F | 40 y | Breast | KIF5B-ALK | Surgical resection | NED (3.5y ) |
| 5 Gupta,et al^[7]^(2019) | F | 50 y | Appendix | KIF5B-ALK | Surgical resection | LTF |
| Lucas,et al^[9]^( 2019) |  |  |  |  |  |  |
| 6 | F | 7 y | CNS | KIF5B-ALK | Surgical resection | NED (1y) |
| 7 | F | 10 y | CNS | KIF5B-ALK | Surgical resection | NED(6 mo) |
| Kashima,et al^[5]^(2021) |  |  |  |  |  |  |
| 8 | F | 45 y | Breast | KIF5B-ALK | Surgical resection | LTF (1 mo) |
| 9 | F | 38 y | Breast | KIF5B-ALK | Surgical resection | LTF (1 mo) |
| 10 Tran,et al^[10]^(2021) | F | 20 y | Mesentery | TRIM33-ALK | Surgical resection | NED (1 y) |
| 11 Jaber,et al^[14]^(2021) | M | 27 y | PNS | KIF5B-ALK | Surgical resection | NED (9 mo) |
| 12 Wolter,et al^15，20]^(2019) | M | 3 y | Soft tissue:  Subglottic mass | KIF5B-ALK | Surgical resection | NED (3 y) |
| 13 Bai,et al^[16]^(2021) | F | 52 y | Lung | EML4-ALK | Surgical resection | NED (5 mo) |
| 14 Rossi,et al^[17，20]^(2021) | F | 11 y | CNS | KIF5B-ALK | Surgical resection | NED (9 mo) |
| 15 Luo,et al^[18]^(2022) | M | 3 y | PNS | KIF5B-ALK | N/A | LTF |
| Osako,et al^[19]^(2022) |  |  |  |  |  |  |
| 16 | F | 30 y | Breast | KIF5B-ALK | Surgical resection | NED (1 y) |
| 17 | F | 40 y | Breast | KIF5B-ALK | Surgical resection | NED (1 y) |
| Kemps, et al^[20]^ (2022) |  |  |  |  |  |  |
| 18 | F | 7 mo | CNS | KIF5B-ALK | Corticosteroids followed by ALK inhibition | ARD (5 mo) |
| 19 | F | 9 mo | CNS | ALK-FISH+ | N/A | LTF |
| 20 | M | 2.5 y | CNS | KIF5B-ALK | Chemotherapy followed by ALK inhibition | NED (16 mo) |
| 21 | F | 3 y | CNS | KIF5B-ALK | Corticosteroids, followed by Surgical resection | NED (12 mo) |
| 22 | F | 3 y | PNS | KIF5B-ALK | Corticosteroids, followed by surgical resection and chemotherapy (vinblastine/prednisone) | ARD (2.5 y) |
| 23 | F | 7 y | CNS | KIF5B-ALK | Chemotherapy  (clofarabine) | NED (6 mo) |
| 24 | M | 11 y | PNS | ALK-FISH+ | Surgical resection | NED (18 y) |
| 25 | M | 12 y | PNS | KIF5B-ALK | Surgical resection followed by  chemotherapy (vinblastine/prednisone) | NED (2.5 y) |
| 25 | F | 13 y | CNS/PNS | KIF5B-ALK | Surgical resection followed by ALK inhibition | ARD (2.5 y) |
| 26 | M | 20 y | PNS | KIF5B-ALK | Not treated yet | Alive with active disease (7 mo) |
| 28 | M | 20 y | CNS | KIF5B-ALK | Surgical resection | NED (10 mo) |
| 29 | F | 6 mo | Skin | KIF5B-ALK | Surgical resection | NED (2 y) |
| 30 | M | 7 mo | Skin | KIF5B-ALK | Surgical resection | NED (13 mo) |
| 31 | F | 21 mo | Skin | KIF5B-ALK | Surgical resection | NED (2 y) |
| 32 | M | 2 y | Soft tissue | KIF5B-ALK | Active monitoring | ARD (14 mo) |
| 33 | F | 3 y | Soft tissue | KIF5B-ALK | Surgical resection | NED (14 mo) |
| 34 | F | 10 y | Skin | KIF5B-ALK | Surgical resection | NED (1 mo) |
| 35 | F | 10 y | Bone | KIF5B-ALK | Surgical resection | NED (5 y) |
| 36 | M | 11 y | Soft tissue | KIF5B-ALK | Surgical resection followed by chemotherapy | SD(15 mo) |
| 37 | M | 17 y | Lung | EML4-ALK | N/A | LTF |
| 38 | F | 41 y | Bone | DCTN1-ALK | ALK inhibition and chemotherapy | ARD (2 y) |

ARD, alive with regressive disease; CNS, central nervous system; F, female; M, male; mo, month; LN, lymph node; LTF, lost to follow-up; N//A, not available; NED, no evidence of disease; PNS, peripheral nervous system; SD, stable disease; y, year.
